# Differential Assembly of Rhizosphere Microbiome and Metabolome in Rice with Contrasting Resistance to Blast Disease

**DOI:** 10.3390/microorganisms13122789

**Published:** 2025-12-08

**Authors:** Jian Wang, Deqiang Li, Daihua Lu, Cheng Chen, Qin Zhang, Rongtao Fu, Fu Huang

**Affiliations:** 1 College of Agriculture, Sichuan Agricultural University, Chengdu 611130, China; 2020101018@stu.sicau.edu.cn (J.W.); ldq122430@163.com (D.L.); 13547420283@163.com (Q.Z.); 2 Key Laboratory of Integrated Pest Management on Crops in Southwest, Institute of Plant Protection, Sichuan Academy of Agricultural Sciences, Ministry of Agriculture, Chengdu 610066, China; ludaihua@scsaas.cn (D.L.); zbschencheng@scsaas.cn (C.C.); furongtao@126.com (R.F.)

**Keywords:** rice blast, microbial community, root exudates, rhizosphere

## Abstract

Rice blast, caused by *Magnaporthe oryzae*, is one of the most devastating diseases threatening global rice production. Although host resistance represents a sustainable control strategy, the underlying mechanisms mediated by the rhizosphere microbiome remain poorly understood. In this study, we selected four rice varieties with varying resistance to blast and demonstrated, through an integrated approach of 16S rRNA/ITS amplicon sequencing, untargeted metabolomics, and soil physicochemical analysis, that the rice genotype reprograms the genotype-root exudate-rhizosphere microbiome system. Results showed that the resistant variety P104 significantly decreased the soil pH while increasing the contents of total nitrogen, ammonium nitrogen, and nitrate nitrogen. On the other hand, the susceptible variety P302 exhibited higher pH and available phosphorus content. Furthermore, the rhizosphere of P104 was enriched with specific beneficial microbes such as *Desulfobacterota*, *Ascomycota*, and *Pseudeurotium*, and activated defense-related metabolic pathways including cysteine and methionine metabolism and phenylpropanoid biosynthesis. In contrast, susceptible varieties showed reduced bacterial diversity and fostered a microecological environment more conducive to pathogen proliferation. Our findings indicate that blast-resistant rice genotypes are associated with a protective rhizosphere microbiome, potentially mediated by alterations in root metabolism, thereby suppressing pathogen establishment. These insights elucidate the underground mechanisms of blast resistance and highlight the potential of microbiome-assisted breeding for sustainable crop protection.

## 1. Introduction

Rice is one of the most vital staple crops, feeding nearly half of the world’s population and playing a critical role in ensuring global food security [1,2]. However, rice blast, caused by the fungal pathogen *Magnaporthe oryzae*, is the most destructive disease affecting rice production, causing annual yield losses of 10–50% worldwide and leading to severe economic and social issues [3,4]. Consequently, preventing the spread and damage of blast is imperative for enhancing rice yields. Current control strategies primarily rely on chemical pesticides and breeding resistant varieties [5]. Although chemical control is effective, the overuse of fungicides poses risks of environmental pollution and human health hazards [6,7]. In contrast, developing and utilizing resistant varieties represents a more sustainable and environmentally friendly strategy. Nevertheless, the resistance mechanisms of host plants against blast, particularly the underground defense mechanisms mediated by plant-soil feedback involving the rhizosphere microbiome, remain inadequately elucidated.

The rhizosphere is a key interface for plant root-microbe interactions and a highly dynamic biochemical hotspot. Plants actively shape their rhizosphere microbial communities through root exudates, which provide nutrients for specific microorganisms, thereby selectively enriching beneficial microbes and constructing a micro-ecosystem conducive to plant health or pathogen suppression [8,9,10]. These microbial communities are regarded as the plant’s second genome and play a central role in regulating host health and disease resistance through various mechanisms such as niche competition, production of antimicrobial compounds, and induction of systemic resistance [11,12,13]. Accumulating evidence indicates that the plant genotype is a key driver in shaping the rhizosphere microbiome. For instance, a foundational study by Edwards et al. [14] on rice demonstrated that different rice genotypes selectively assemble distinct bacterial communities in their rhizospheres, with genetic effects being most pronounced in the root compartment. This genotype-dependent assembly suggests that rice varieties can actively shape their microbial partners from the available soil pool. Meanwhile, plants of different genotypes will specifically recruit microorganisms with potential in enhancing disease resistance. Plants can selectively enrich beneficial microorganisms to inhibit soil-borne pathogens [15]. Among various beneficial microbes, *Bacillus* species have attracted significant attention due to their strong environmental adaptability and multiple probiotic functions, including the production of antimicrobial substances and induction of plant systemic resistance against various pathogens [16]. For example, *Bacillus velezensis* FZB42 can inhibit the emergence of pathogens in multiple plants through its ability to produce antimicrobial compounds, while *Bacillus velezensis* SQR9 can induce systemic resistance to prevent fungal pathogen infections [17,18,19].

Despite these significant advances, most studies have focused on a single microbial domain or have failed to systematically link structural changes in the microbiome with plant metabolic responses. Specifically, how the overall response patterns of the rhizosphere microbial community differ among rice varieties with varying levels of blast resistance, and how these patterns are coupled with changes in root metabolomes and soil physicochemical properties, remains a critical knowledge gap. Understanding these multi-interface interactions is essential for comprehensively revealing the underground mechanisms of plant disease resistance.

Therefore, this study selected four rice varieties with different resistance levels to blast. By integrating 16S rRNA/ITS amplicon sequencing, untargeted metabolomics, and soil physicochemical analysis, we aim to elucidate the interactions among rice genotypes, microbiomes, and metabolomes. This work provides new insights into the underground mechanisms of blast resistance and lays a theoretical foundation for future development of microbiome-based crop protection strategies.

## 2. Materials and Methods

### 2.1. Plant Materials and Experimental Design

The rice seeds used in this study were obtained from the Plant Protection Institute, Sichuan Academy of Agricultural Sciences. The varieties were P104 (highly resistant), P206 (moderately resistant), P309 (moderately susceptible), and P302 (susceptible). The experiment was conducted in 2025 at the experimental base of the Sichuan Academy of Agricultural Sciences. Rice seeds were conventionally nursery-grown in paddy fields, and seedlings were manually transplanted at the 3–4 leaf stage (two seedlings per hill). The field was divided into 12 plots, each separated by 1 m, with each plot covering an area of 4 m^2^ (2 m length × 2 m width). The four rice varieties with different blast resistances were planted in a randomized complete block design with three replicates per variety. Each plot contained 6 rows with 6 hills per row, and the spacing between hills was 20 cm. Each plot contained 36 seedlings, totaling 432 seedlings for the entire experiment. Pest and disease management was performed according to standard practices during the growing season.

### 2.2. Soil Collection and Physicochemical Analysis

After the rice plants reached maturity in each plot, the root systems were carefully uprooted, and loosely adhering soil was removed. Soil tightly adhering to the roots was collected as rhizosphere soil, placed into sterile plastic bags, and stored at 4 °C for subsequent microbial analysis. Additionally, bulk soil from the 0–15 cm surface layer was collected from each plot. After removing stones, weeds, roots, and other debris, the soil was sealed in plastic bags for physicochemical analysis. Soil pH was measured using a pH meter (FE28, METTLER-TOLEDO, Columbus, OH, USA). Soil organic carbon (OC) content was determined following the method described by Cui et al. [20]. The total nitrogen (TN) content was measured according to Xiong et al. [21]. The total phosphorus (TP) content was analyzed using the method of Li et al. [22]. The total potassium (TK) content was determined according to Zavyalova et al. [23]. The hydrolyzable nitrogen (HN) content was assessed following Roberts et al. [24]. The available phosphorus (AP) and available potassium (AK) contents were measured according to Zhang et al. [25]. The ammonium nitrogen (NH_4_^+^-N), nitrate nitrogen (NO_3_^−^-N), and nitrite nitrogen (NO_2_^−^-N) contents were determined using the method described by Liu et al. [26]. All analyses were performed with three replicates.

### 2.3. Analysis of Rhizosphere Soil Microorganisms

Microbial DNA extraction and 16S rRNA/ITS sequencing were performed using standard protocols. DNA was extracted from the rhizosphere soil samples using a soil DNA extraction kit. DNA samples were diluted to 1 ng/μL, and purity was checked via 1% (*w*/*v*) agarose gel electrophoresis. DNA quality was assessed using a NanoDrop One spectrophotometer (Thermo Fisher Scientific, Wilmington, DE, USA). The V3–V4 hypervariable region of the bacterial 16S rRNA gene was amplified using primers 341F (CCTAYGGGRBGCASCAG) and 806R (GGACTACNNGGGTATCTAAT). The fungal ITS1-5F region was amplified using primers 1737F (GGAAGTAAAAGTCGTAACAAGG) and 2043R (GCTGCGTTCTTCATCGATGC) [27,28]. Additionally, the nitrogen-fixing bacteria *nifH* gene fragment was amplified using primers nifHf (AAAGGYGGWATCGGYAARTCCACCAC) and nifHR (TTGTTSGCSGCRTACATSGCCATCAT) [29]. Quantitative PCR (qPCR) was conducted following the procedure outlined by Xiong et al. [30]. After library construction, quantification was performed using the Qubit and qPCR. Qualified libraries were sequenced on an Illumina platform by Beijing Novogene Technology Co., Ltd. (Beijing, China). After removing the barcode and primer sequences, reads from each sample were assembled using FLASH (version 1.2.11) [31] to generate raw tags. Raw tags were quality-filtered using Fastp software (version 0.23.1) to obtain clean tags [32]. Chimeric sequences were identified and removed by comparing tag sequences with reference databases, resulting in effective tags [33]. Effective tags were denoised using the DADA2 module in QIIME2 software (version 2024.5) to obtain amplicon sequence variants (ASVs) [34]. Taxonomic annotation was performed in QIIME2 using the Silva 138.1 database for 16S rRNA and the UNITE v9.0 database for ITS. Microbial alpha and beta diversity analyses were conducted using QIIME2 software (version 2024.5). PICRUSt (version 1.1.4) was used to predict metagenomic functions based on marker genes.

### 2.4. Analysis of Root Metabolites

Roots from each rice variety were collected, rinsed with distilled water, placed into Eppendorf tubes (Eppendorf AG, Hamburg, Germany), and ground into powder using liquid nitrogen. A 100 mg aliquot of ground tissue was used for metabolite extraction. Then, 500 μL of an 80% methanol aqueous solution was added to the tube, followed by vortexing and incubation in an ice bath for 5 min. The mixture was centrifuged at 15,000× *g* and 4 °C for 20 min using a refrigerated centrifuge (D3024R, Scilogex, Rocky Hill, CT, USA). The supernatant was collected and the methanol content was diluted to 53%. The solution was centrifuged again under the same conditions. The final supernatant was collected for LC-MS analysis [35]. For the specific operation process of LC-MS analysis, please refer to the Methods Section in the Supplementary Materials.

### 2.5. Statistical Analysis

Data processing was performed on a Linux operating system (CentOS version 6.6) using R (versions 3.4.3 and 4.0.3) and Python (versions 2.7.6 and 3.5.0). Statistical significance between samples was analyzed using Duncan’s multiple range test and independent samples *t*-test in IBM SPSS Statistics 26.0. The *p* value < 0.05 was considered statistically significant.

## 3. Results

### 3.1. Differences in Soil Physicochemical Properties

Table 1 shows the differences in soil physicochemical properties after cultivating the different rice varieties. The pH value in the P302 group (6.24 ± 0.03) was significantly higher than in the other groups, while the P104 group had the significantly lowest pH (5.90 ± 0.02) (*p* < 0.05). No significant differences were observed in soil OC content among groups. For soil TN content, the P104 group had significantly higher TN than the P206 and P302 groups, while the P302 group had significantly lower TN than all other groups (*p* < 0.05). For soil TP content, the P302 and P309 groups had significantly higher TP than the P104 and P206 groups (*p* < 0.05). For soil TK content, the P309 group had significantly higher TK than the other groups (*p* < 0.05), while no significant differences were found among the P104, P206, and P302 groups. For soil HN content, the P206 group had significantly lower HN than the other groups (*p* < 0.05), while no significant differences were observed among the P104, P302, and P309 groups. For soil AP content, the P302 group had significantly higher AP than the other groups, while the P104 and P309 groups had significantly lower AP (*p* < 0.05). For soil AK content, the P104 and P302 groups had significantly higher AK than the P206 and P309 groups (*p* < 0.05). For soil NH_4_^+^-N content, the P104 and P309 groups had significantly higher NH_4_^+^-N than the P206 and P302 groups (*p* < 0.05). For soil NO_3_^−^-N content, the P104 group had significantly higher NO_3_^−^-N than the other groups, while the P206 group had significantly lower NO_3_^−^-N (*p* < 0.05). For soil NO_2_^−^-N content, the P206 group had significantly lower NO_2_^−^-N than the other groups (*p* < 0.05), while no significant differences were found among the P104, P302, and P309 groups.

### 3.2. Changes in Rhizosphere Soil Microbial Communities

#### 3.2.1. Sequencing Data Analysis

Rhizosphere soil microorganisms from the four rice varieties with different blast resistance levels were analyzed via 16S rRNA and ITS sequencing. As shown in Figure S1, after sequencing reads exceeded 20,000, the rarefaction curves for all bacterial and fungal communities gradually plateaued, indicating sufficient sequencing depth to capture the overall structure of the rice rhizosphere microbiota. After quality control and filtering, the average effective reads used for subsequent analysis were 81,601 for bacteria, 95,637 for fungi, and 181,934 for nitrogen-fixing bacteria, respectively.

#### 3.2.2. Microbial Composition and Abundance

**Figure 1:**
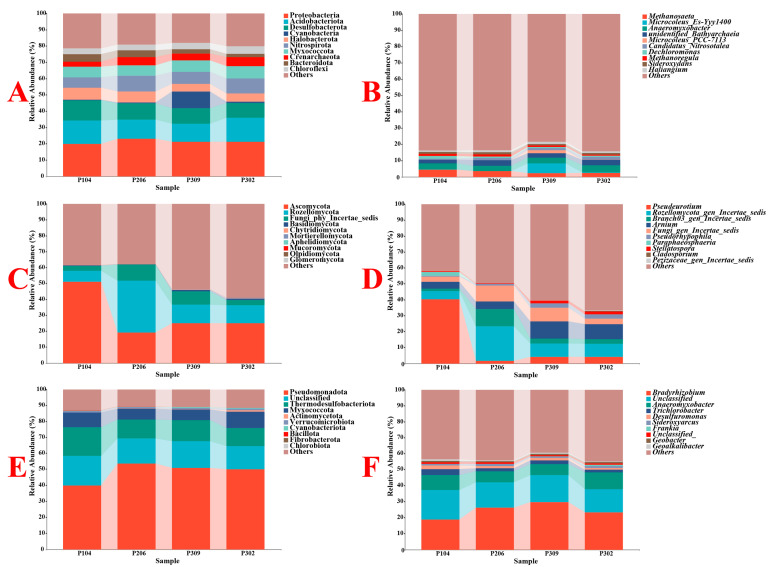
Abundance of the top ten bacteria (**A**,**B**), fungi (**C**,**D**), and nitrogen-fixing bacteria (**E**,**F**) in rice rhizosphere soil at the phylum and genus levels.

Across all rhizosphere soil samples, 69 bacterial phyla, 157 classes, 313 orders, 415 families, and 680 genera were identified. For fungi, 14 phyla, 40 classes, 84 orders, 169 families, and 249 genera were identified. For nitrogen-fixing bacteria, 19 phyla, 39 classes, 77 orders, 129 families, and 239 genera were identified. The changes in the top ten most abundant phyla and genera for bacteria, fungi, and nitrogen-fixing bacteria are compared in Figure 1.

At the bacterial phylum level, *Proteobacteria* was the most abundant (21.36%), followed by *Acidobacteriota* (12.89%) and *Desulfobacterota* (12.12%). The abundance of *Desulfobacterota* in the P104 group was significantly higher than in other groups (*p* < 0.05). At the genus level, *Anaeromyxobacter* was the most abundant (3.74%), with its abundance in the P302 group being significantly higher than in the P206 and P309 groups (*p* < 0.05). This was followed by *Methanosaeta* (3.25%) and unidentified *Bathyarchaeia* (2.92%). Interestingly, the abundance of *Microcoleus* Es-Yyy1400 in the P309 group was significantly higher than in other groups (*p* < 0.05).

At the fungal phylum level, *Ascomycota* was the most abundant (30.06%). Its abundance in group P104 was significantly higher, and in group P206 significantly lower, than in other groups (*p* < 0.05). This was followed by *Rozellomycota* (15.59%), which showed significantly higher abundance in group P206 and significantly lower abundance in group P104 compared to others (*p* < 0.05). The third most abundant was *Fungi phy Incertae sedis* (6.25%), with significantly higher abundance in groups P206 and P309 compared to groups P104 and P302 (*p* < 0.05). At the genus level, *Pseudeurotium* was the most abundant (12.48%). Notably, its abundance in group P104 was significantly higher, and in group P206 significantly lower, than in other groups (*p* < 0.05). This was followed by *Rozellomycota gen Incertae sedis* (10.87%), which showed significantly higher abundance in group P206 and significantly lower abundance in group P104 (*p* < 0.05). The third most abundant was *Arnium* (7.40%), with significantly higher abundance in groups P309 and P302 compared to groups P104 and P206 (*p* < 0.05).

Among nitrogen-fixing bacteria, *Pseudomonadota* was the most abundant phylum (48.47%), with a significantly higher abundance in the P206 group and a significantly lower abundance in the P104 group (*p* < 0.05). This was followed by *Unclassified* (16.47%), which had a significantly higher abundance in the P104 group than in the P206 and P302 groups (*p* < 0.05). The third most abundant was *Thermodesulfobacteriota* (13.55%), showing a significantly higher abundance in the P104 group and a significantly lower abundance in the P302 group (*p* < 0.05). At the genus level, *Bradyrhizobium* was the most abundant (24.51%), with a significantly higher abundance in the P309 group and a significantly lower abundance in the P104 group (*p* < 0.05). This was followed by *Unclassified* (16.34%), which had a significantly higher abundance in the P104 group than in the P206 and P302 groups (*p* < 0.05). The third most abundant was *Anaeromyxobacter* (8.31%), with a significantly higher abundance in the P302 group (*p* < 0.05).

#### 3.2.3. Alpha Diversity of Microbial Communities

**Figure 2:**
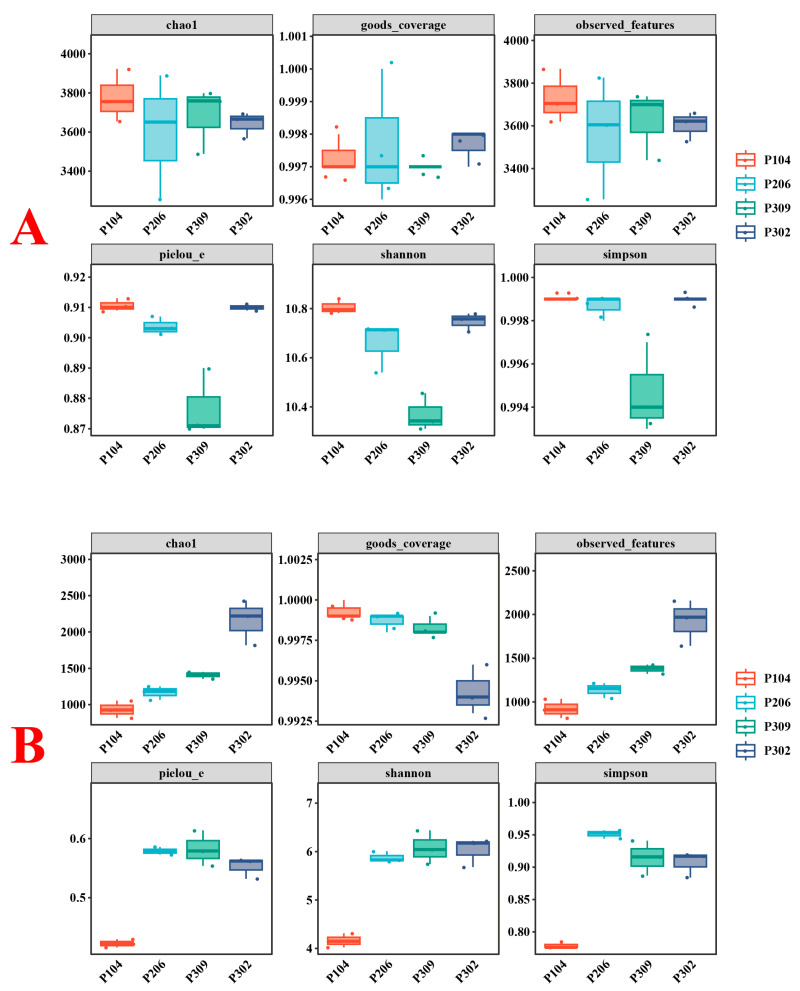
Box plots of α-diversity indices for bacteria (**A**) and fungi (**B**) in rhizosphere soil of rice.

Figure 2 shows the differences in alpha diversity indices for rhizosphere soil bacteria and fungi. For bacteria, the P309 group had significantly lower Pielou e, Shannon, and Simpson indices than the other groups (*p* < 0.05). Interestingly, more pronounced differences were observed in fungal diversity. The P302 group had significantly higher Chao1 and observed features indices but a significantly lower Goods coverage index than the other groups (*p* < 0.05). Additionally, the P104 group had a significantly higher observed features index than the P309 group (*p* < 0.05). The P104 group also had significantly higher Pielou’s evenness and Shannon indices than the P309 and P302 groups (*p* < 0.05). For nitrogen-fixing bacteria, the P104 group had a significantly higher Pielou’s evenness index than the other groups, and significantly higher Shannon and observed features indices than the P309 group (Figure S2).

#### 3.2.4. Structural Changes in Rhizosphere Soil Microbial Communities

**Figure 3:**
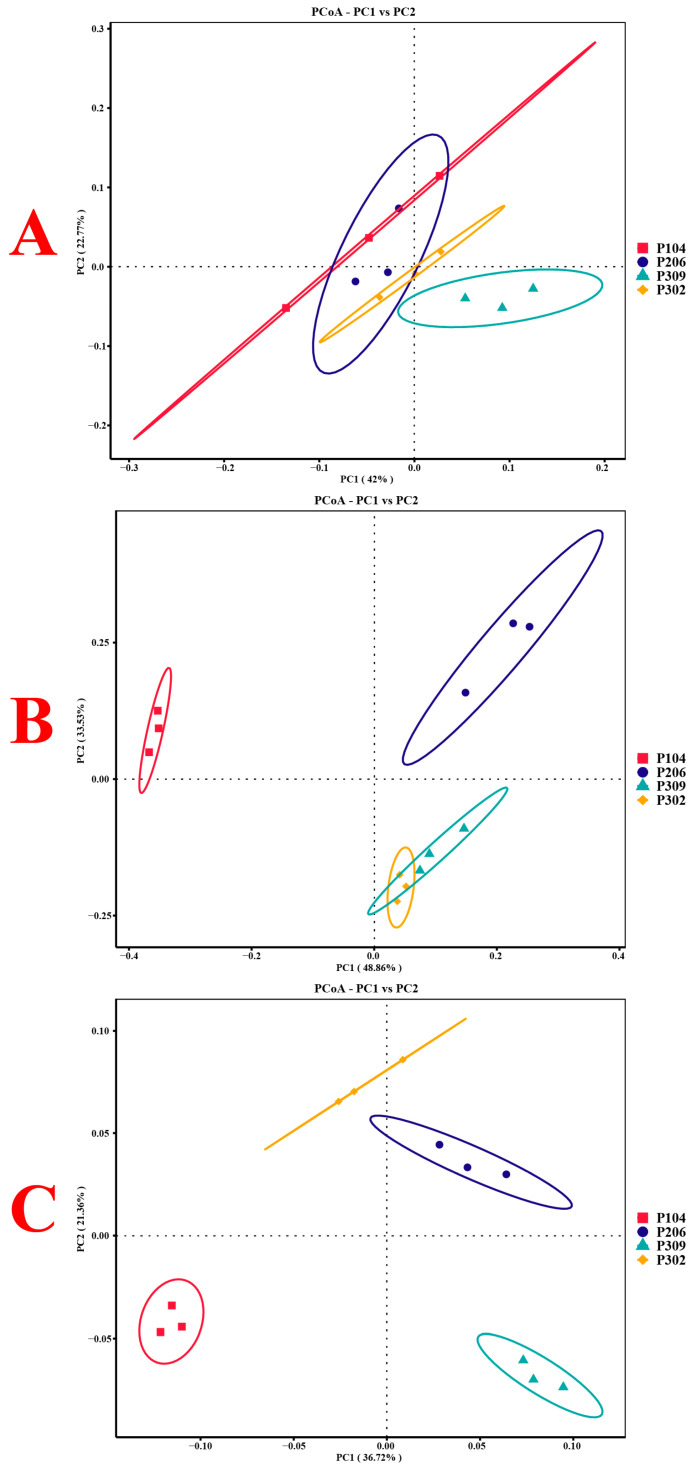
PCoA plots of β-diversity for rhizosphere soil bacteria (**A**), fungi (**B**), and nitrogen-fixing bacteria (**C**) in rice. The circles are confidence ellipses drawn around the sample points of each processing group.

**Figure 4:**
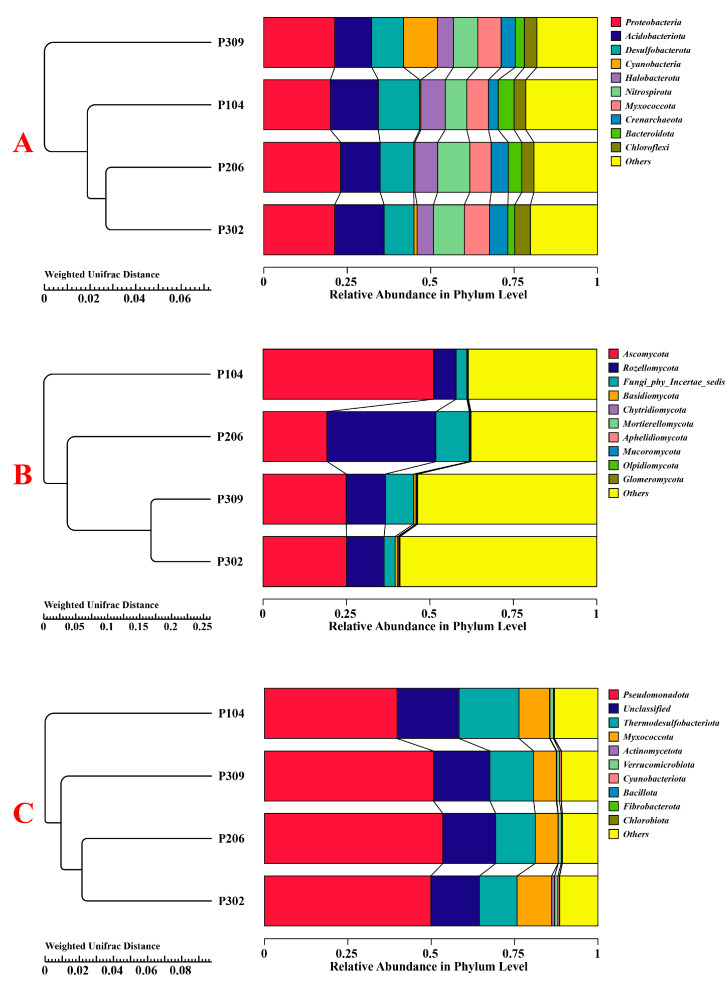
Changes in bacterial (**A**), fungal (**B**), and nitrogen-fixing bacterial (**C**) communities in rice rhizosphere soil based on weighted UniFrac distance.

Principal coordinate analysis (PCoA) based on weighted UniFrac distance was used to analyze community structural changes in bacteria, fungi, and nitrogen-fixing bacteria (Figure 3). The results indicated relatively small structural changes among bacterial communities, whereas larger changes were observed for fungal and nitrogen-fixing bacteria communities. Based on weighted UniFrac distance, the P104 group exhibited the largest structural variation in fungal communities, while the P309 and P302 groups had the most similar structures (Figure 4). For nitrogen-fixing bacteria, the P104 group also showed the largest structural variation, while the P206 and P302 groups were most similar.

#### 3.2.5. Functional Changes in Rhizosphere Soil Microorganisms

Analysis based on the COG database revealed that the functional levels of COG0438 (a glycosyltransferase involved in cell wall biosynthesis), COG2204 (a DNA-binding transcriptional response regulator), and COG0845 (a multidrug efflux pump subunit Acr) in the P309 group were significantly lower than those in the other groups (*p* < 0.05). In contrast, the functional level of COG1028 (an NAD(P)-dependent dehydrogenase) in the P302 group was significantly higher than that in the other groups (*p* < 0.05). Analysis based on the KO database showed that the functional level of K06147 (an ATP-binding cassette) in the P309 group was significantly higher than that in the other groups (*p* < 0.05). Additionally, the functional level of K00059 (a 3-oxoacyl-[acyl-carrier protein] reductase) in the P302 group was significantly higher than that in the other groups (*p* < 0.05). Pathway database analysis indicated that the functional levels of P42-PWY (an incomplete reductive TCA cycle), PWY-5101 (L-isoleucine biosynthesis II), VALSYN-PWY (L-valine biosynthesis), ILEUSYN-PWY (L-isoleucine biosynthesis I), BRANCHED-CHAIN-AA-SYN-PWY (the superpathway of branched amino acid biosynthesis), and PWY-5103 (L-isoleucine biosynthesis III) in the P309 group were significantly lower than those in the other groups (*p* < 0.05). The functional level of PWY-3781 (aerobic respiration I) in the P302 group was significantly higher than that in the other groups (*p* < 0.05).

Guild analysis revealed that the abundance of Unassigned and Undefined Saprotroph in the P104 group was significantly lower than in other groups (*p* < 0.05). The P302 group had a significantly higher abundance of guilds such as Animal Pathogen-Endophyte-Fungal Parasite-Lichen Parasite-Plant Pathogen-Wood Saprotroph and *Clavicipitaceous* Endophyte-Plant Pathogen compared to other groups (*p* < 0.05). Furthermore, the abundances of Dung Saprotroph-Undefined Saprotroph and *Clavicipitaceous* Endophyte-Plant Pathogen in the P104 and P206 groups were significantly lower than in the P309 and P302 groups (*p* < 0.05).

### 3.3. Differences in Root Metabolites

Volcano plots visually display the changes in metabolites among the roots of different rice varieties (Figures S3 and S4). Under the criteria of VIP > 1.6 and *p* < 0.05, the top five most significant differential metabolites between group comparisons were identified (Table S1).

Between the P104 and P206 groups, 135 metabolites were up-regulated and 121 were down-regulated in positive ion mode, while 140 were up-regulated and 20 were down-regulated in negative ion mode. Metabolites such as 3alpha-Isobutyryloxytropane and 2-hydroxyoctanoic acid changed significantly (*p* < 0.05). Between the P104 and P309 groups, 146 metabolites were up-regulated and 179 were down-regulated in positive ion mode, while 100 were up-regulated and 66 were down-regulated in negative ion mode. Metabolites like Kyotorphin and 5-methyluridine changed significantly (*p* < 0.05). Between the P104 and P302 groups, 418 metabolites were up-regulated and 309 were down-regulated in positive ion mode, while 188 were up-regulated and 166 were down-regulated in negative ion mode. Metabolites such as Lysylasparagine and Caffeic acid 3-glucoside changed significantly (*p* < 0.05). Between the P206 and P302 groups, 398 metabolites were up-regulated and 363 were down-regulated in positive ion mode, while 113 were up-regulated and 256 were down-regulated in negative ion mode. Metabolites like Val-Glu-Leu and 5-Ethoxythiazole changed significantly (*p* < 0.05). Between the P206 and P309 groups, 110 metabolites were up-regulated and 203 were down-regulated in positive ion mode, while 43 were up-regulated and 129 were down-regulated in negative ion mode. Metabolites such as 9-Aminononanoic acid and Allantoin changed significantly (*p* < 0.05). Between the P302 and P309 groups, 281 metabolites were up-regulated and 396 were down-regulated in positive ion mode, while 210 were up-regulated and 148 were down-regulated in negative ion mode. Differential metabolites like Polanrazine E and 5,6-Dimethoxy-3-(4′-methoxyphenylmethyl)phthalide changed significantly (*p* < 0.05).

**Figure 5:**
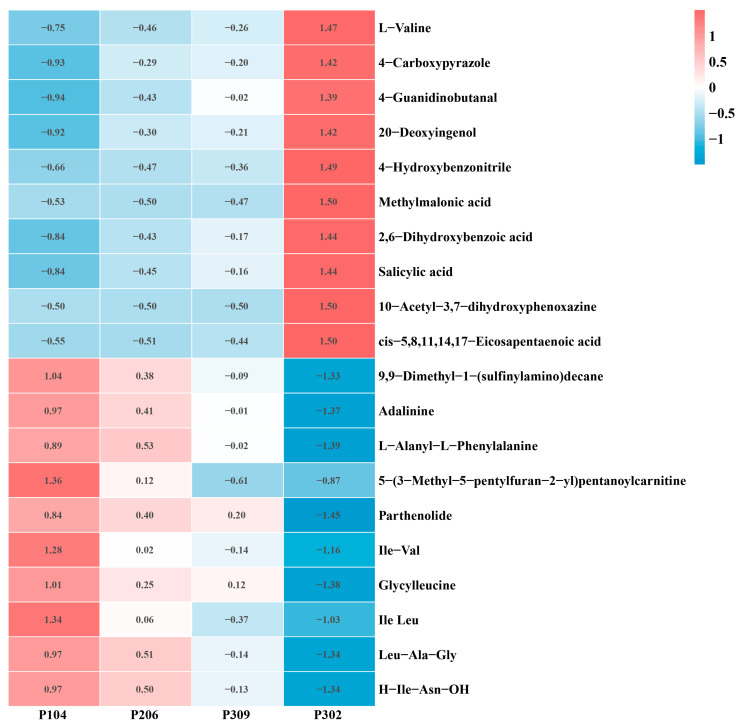
Changes in the top 20 differentially expressed metabolites with the greatest continuous expression differences across rice varieties.

Analysis of differential metabolites across P104, P206, P309, and P302 groups revealed that, in positive ion mode, the expression levels of 22 differential metabolites (e.g., L-Valine, 4-Carboxypyrazole, 4-Guanidinobutanal) significantly increased as blast resistance decreased (*p* < 0.05). Conversely, the expression levels of 24 differential metabolites (e.g., 9,9-Dimethyl-1-(sulfinylamino)decane, Adalinine, L-Alanyl-L-Phenylalanine) significantly decreased with decreasing resistance. In negative ion mode, the expression levels of 19 differential metabolites (e.g., Methylmalonic acid, 2,6-Dihydroxybenzoic acid, Salicylic acid) significantly increased as resistance declined (*p* < 0.05). Meanwhile, the expression levels of 27 differential metabolites (e.g., Ile-Val, Glycylleucine, Ile-Leu) significantly decreased with decreasing resistance (*p* < 0.05). Figure 5 shows the changes in the top 20 differential metabolites with the largest differences in expression among the groups.

**Figure 6:**
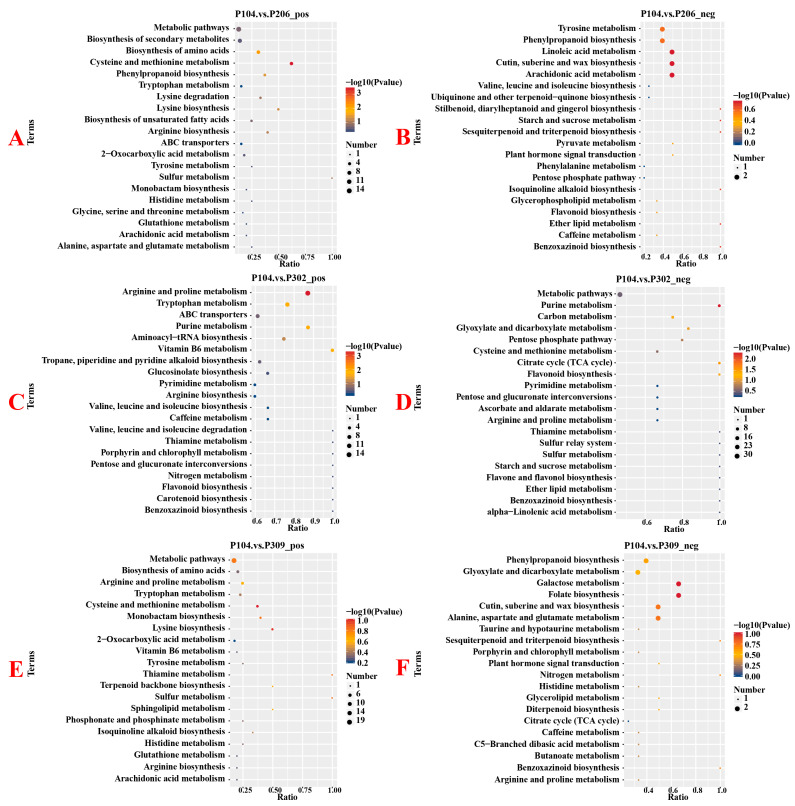
KEGG enrichment analysis bubble plots of differential metabolites in rice root systems under positive and negative ion conditions. (**A**) KEGG enrichment analysis bubble chart for the P104 and P206 groups under positive ion conditions; (**B**) KEGG enrichment analysis bubble chart for the P104 and P206 groups under negative ion conditions; (**C**) KEGG enrichment analysis bubble chart for the P104 and P302 groups under positive ion conditions; (**D**) KEGG enrichment analysis bubble plot for P104 and P302 groups under negative ion conditions; (**E**) KEGG enrichment analysis bubble plot for P104 and P309 groups under positive ion conditions; (**F**) KEGG enrichment analysis bubble plot for P104 and P309 groups under negative ion conditions.

KEGG enrichment analysis showed that, compared to the P206 group, metabolic pathways such as Cysteine and methionine metabolism, Biosynthesis of amino acids, and Phenylpropanoid biosynthesis were significantly affected in the P104 group (Figure 6). Compared to the P302 group, pathways such as Arginine and proline metabolism and Purine metabolism were significantly affected in the P104 group. However, no significant changes were detected in metabolic pathways between the P104 and P309 groups.

### 3.4. Correlation Analysis

**Figure 7:**
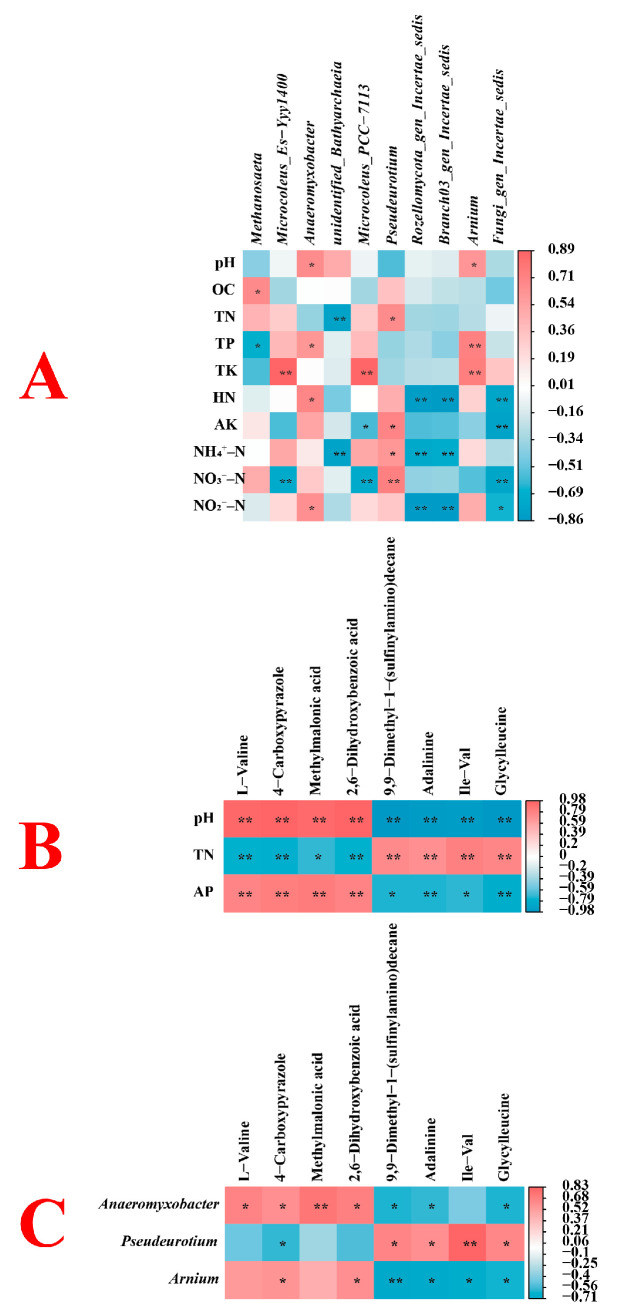
Analysis of the correlation between soil physicochemical properties, rhizosphere soil microorganisms, and root metabolites in rice soil. (**A**) Correlation diagram between soil physicochemical properties and rhizosphere soil microorganisms. (**B**) Correlation diagram between soil physicochemical properties and root metabolites. (**C**) Correlation diagram between rhizosphere soil microorganisms and root metabolites. One asterisk (*) indicates *p* < 0.05, two asterisks (**) indicate *p* < 0.01.

Figure 7 presents Pearson correlation analysis among soil physicochemical properties, the top ten most abundant microorganisms, and consecutively significantly changed differential metabolites.

In the relationship between soil properties and microbes, *Pseudeurotium* showed a significant positive correlation with soil NO_3_^−^-N content, while *Arnum* was significantly positively correlated with soil TP and TK contents (*p* < 0.01) (Figure 7A). Unidentified *Bathyarchaeia* was significantly negatively correlated with soil TN and NH_4_^+^-N contents, and *Fungi gen Incertae sedis* was significantly negatively correlated with soil HN, AK, and NO_3_^−^-N contents (*p* < 0.01). Both *Rozellomycota gen Incertae sedis* and *Branch03 gen Incertae sedis* were significantly negatively correlated with soil HN, NH_4_^+^-N, and NO_2_^−^-N contents (*p* < 0.05). Furthermore, *Microcoleus Es-Yyy1400* and *Microcoleus PCC-7113* were significantly positively correlated with soil TK content but significantly negatively correlated with soil NO_3_^−^-N content (*p* < 0.01).

Regarding the relationship between soil properties and differential metabolites, pH was significantly positively correlated with L-Valine, 4-Carboxypyrazole, Methylmalonic acid, and 2,6-Dihydroxybenzoic acid, but significantly negatively correlated with 9,9-Dimethyl-1-(sulfinylamino)decane, Adalinine, Ile-Val, and Glycylleucine (*p* < 0.01) (Figure 7B). Soil TN content was significantly negatively correlated with L-Valine, 4-Carboxypyrazole, and 2,6-Dihydroxybenzoic acid, but significantly positively correlated with 9,9-Dimethyl-1-(sulfinylamino)decane, Adalinine, Ile-Val, and Glycylleucine (*p* < 0.01). Soil AP content was significantly positively correlated with L-Valine, 4-Carboxypyrazole, Methylmalonic acid, and 2,6-Dihydroxybenzoic acid, but significantly negatively correlated with Adalinine and Glycylleucine (*p* < 0.01).

In the microbe-metabolite relationships, *Anaeromyxobacter* was significantly positively correlated with Methylmalonic acid (*p* < 0.01) (Figure 7C). *Pseudeurotium* was significantly positively correlated with Ile-Val (*p* < 0.01). Additionally, *Arnum* was significantly negatively correlated with 9,9-Dimethyl-1-(sulfinylamino)decane (*p* < 0.01).

## 4. Discussion

The composition of soil microorganisms is closely related to the physicochemical environment of the soil and significantly influences crop growth, development, and disease resistance [36,37,38,39,40]. The interaction between plant rhizosphere microbes and the host is recognized as crucial for plant health, enhancing stress resistance through synergistic effects between beneficial microbial communities and plant traits [41,42,43]. Our study, integrating a systematic analysis of soil properties, rhizosphere microbiota, and root metabolomes, reveals significant differences in the rhizosphere micro-ecosystems of rice varieties with contrasting blast resistance. These differences are reflected not only in the composition and diversity of the rhizosphere microbial community, but are also tightly coupled with soil nutrient cycling and plant metabolic responses, providing new perspectives for understanding the underground mechanisms of rice blast resistance.

Our findings show that rice varieties with different resistance levels significantly alter the physicochemical properties of the rhizosphere soil. The resistant variety P104 is associated with a lower soil pH and higher TN, NH_4_^+^-N, and NO_3_^−^-N contents, while the susceptible variety P302 has a higher pH and AP content. These differences in physicochemical factors may directly or indirectly shape specific microbial community structures in the rhizosphere by influencing the survival environment and activity of microorganisms. For instance, a lower pH environment might favor the enrichment of certain functional microbes, consistent with previous studies identifying soil pH as a key driver of microbial community structure [44,45,46]. Furthermore, the higher NO_3_^−^-N content in the P104 rhizosphere might enhance the activity of nitrifying bacteria, potentially indirectly boosting plant immunity by regulating nitrogen metabolism [47].

Regarding the rhizosphere soil microorganisms, the resistant variety P104 displayed a unique microbial assembly pattern. We observed that resistant rice varieties might actively shape a rhizosphere microenvironment beneficial to their health by altering root exudation patterns. Studies have shown that plants under pathogen stress can recruit beneficial microbes via root exudates to assist in disease resistance [48,49]. For example, Berendsen et al. [50] found that *Arabidopsis thaliana*, upon infection with downy mildew, specifically enriched a beneficial bacterial consortium comprising *Xanthomonas*, *Stenotrophomonas*, and *Microbacterium* in its rhizosphere, inducing systemic resistance and promoting plant growth. In our study, the significant enrichment of *Desulfobacterota*, *Ascomycota*, and *Pseudeurotium* in the P104 rhizosphere might represent a specific response of resistant rice to blast stress, actively recruiting beneficial microbial taxa. Root exudates play a crucial role as key mediators in shaping the rhizosphere microbiome [51]. Yuan et al. [52] found that *Arabidopsis thaliana*, upon leaf infection by *Pseudomonas syringae*, significantly increased the secretion of amino acids (AAs) and long-chain organic acids (LCOAs) in root exudates, while decreasing sugars and short-chain organic acids (SCOAs). Exogenous application of a mixture of AAs and LCOAs to the soil successfully mimicked the pathogen-induced soil suppressiveness, significantly reducing disease incidence in subsequent plants. This aligns well with our findings, where defense-related root metabolic pathways such as amino acid metabolism and phenylpropanoid biosynthesis were significantly activated in the resistant variety P104. This suggests that rice may undergo similar metabolic reprogramming, adjusting its root exudation profile to selectively filter and enrich rhizosphere microbes capable of providing protective functions. It is worth noting that our metabolomic analysis was performed on root tissues rather than on rhizosphere soil extracts. While root metabolic profiles are often correlated with root exudate composition, future studies directly profiling rhizosphere metabolites are needed to confirm the role of exudates in microbiome assembly.

The bacterial diversity of the susceptible variety P302 did not undergo significant changes. However, in the fungal community, its Chao1 index and Observed features index were the highest, while its Good’s coverage index was the lowest. The higher Chao1 index coupled with lower Good’s coverage in P302 suggests a high richness of fungal species, including many rare taxa. This pattern may reflect a more heterogeneous fungal community structure in the susceptible variety. PCoA analysis indicated that the rhizosphere fungal and nitrogen-fixing bacteria communities of the resistant variety P104 were clearly separated from those of other varieties, particularly the susceptible ones, emphasizing the strong filtering capacity of the rice genotype on specific microbial domains [14,53]. More importantly, this plant genotype-driven microbial community differentiation can form a soil-borne legacy effect [8]. Plants subjected to multiple generations of pathogen stress can retain a microbial community structure in the soil that benefits subsequent disease resistance, protecting offspring plants from infection. The specific microbial combination in the rhizosphere of the resistant varieties in our study likely constitutes a beneficial underground resistance trait.

Functional prediction analysis provided deeper insights into the ecological functions of the microbial communities. The susceptible variety P309 showed a significantly reduced functional potential for multiple COG functions related to cell wall synthesis, transcriptional regulation, and drug efflux, as well as several branched-chain amino acid biosynthesis pathways. This might indicate weaker metabolic flexibility and adaptability of its rhizosphere microbiome in response to biotic or abiotic stresses. Conversely, functions related to aerobic respiration and dehydrogenase activity were more active in the susceptible variety P302, possibly reflecting a different energy metabolic state in its rhizosphere microenvironment. Crucially, Guild functional prediction indicated that the P302 rhizosphere was enriched with various potential plant and animal pathogen functional guilds, suggesting that its rhizosphere may foster a microenvironment more conducive to pathogen proliferation.

At the plant metabolic response level, we identified a series of key differential metabolites that were significantly positively or negatively correlated with rice blast resistance. As resistance decreased, the expression levels of metabolites such as L-Valine, Methylmalonic acid, and Salicylic acid increased, while the levels of various dipeptides and special metabolites decreased. Salicylic acid is a key signaling molecule for systemic acquired resistance in plants its accumulation in susceptible roots might be a passive pathological response or indicative of dysregulation in defense signaling [54,55,56]. Interestingly, we observed that certain defense-related metabolites, such as salicylic acid and 2,6-dihydroxybenzoic acid, were more abundant in susceptible varieties. This may reflect a heightened but ineffective defense response in these plants, where pathogen pressure triggers metabolic activation without successful containment of the disease. Alternatively, it is possible that the pathogen itself manipulates host metabolism to enhance its own fitness. Further mechanistic studies are needed to dissect the causal relationships behind these metabolic shifts. Conversely, the reduction of dipeptides in resistant varieties’ roots suggests that resistant varieties might possess more efficient protein turnover and amino acid recycling, redirecting resources towards the synthesis of resistance-related metabolites [57]. KEGG enrichment analysis found that pathways closely associated with disease resistance, such as cysteine and methionine metabolism, phenylpropanoid biosynthesis, and arginine and proline metabolism, were significantly activated in the resistant variety P104. These pathways play central roles in the synthesis of plant secondary metabolites and antioxidant defense [58].

Finally, correlation network analysis closely linked the soil environment, microbes, and plant metabolism. For example, the key fungal genus *Pseudeurotium* was positively correlated with soil NO_3_^−^-N content and the beneficial dipeptide Ile-Val, suggesting its potential role as a beneficial microbe in the P104 rhizosphere, playing a positive role in nitrogen utilization and plant health. The significant correlation between *Anaeromyxobacter* and Methylmalonic acid reveals potential microbe-metabolite interactions regulating the rhizosphere microenvironment.

In summary, this study demonstrates that rice varieties with different blast resistance levels differentially shape the rhizosphere soil environment through root activity and selectively enrich beneficial microbial communities with specific structures and functions by adjusting root metabolite exudation profiles. This synergistic interaction network of genotype-root metabolism-rhizosphere microbiome collectively constitutes an important underground mechanism for rice blast resistance and may establish a beneficial soil legacy effect. Future research should focus on identifying key functional factors from these correlated microbes and metabolites, validating their causal roles in enhancing rice blast resistance through isolation, synthetic community construction, and inoculation experiments, thereby providing a theoretical basis for developing novel, rhizosphere microbiome-based, eco-friendly disease control strategies.

## 5. Conclusions

This study systematically deciphered the microecological regulatory mechanisms in the rice rhizosphere underlying blast resistance by integrating 16S rRNA/ITS amplicon sequencing, untargeted metabolomics, and soil physicochemical analysis. Our results demonstrate that rice genotypes with varying resistance levels reshape the rhizosphere microenvironment by altering soil properties, microbial community structure, and root metabolites. The resistant variety P104 recruited a rhizosphere microbiome dominated by beneficial taxa, exhibiting higher functional potential in nutrient cycling and stress adaptation. In contrast, susceptible varieties hosted microbial communities with higher pathogen abundance and reduced metabolic activity. Importantly, the activation of root defense-related metabolic pathways in disease-resistant rice, coupled with the reorganization of the rhizosphere microbial community structure, reveals a potential underground coordinated disease resistance strategy. These genotype-driven rhizosphere changes may establish a soil legacy effect, potentially enhancing plant ability to resist pathogen invasion over time. This research elucidates the interactions among rice genotype, microbiota, and metabolome, clarifying the underground mechanisms of blast resistance and laying a theoretical foundation for future development of microbiome-based crop protection strategies.

## Figures and Tables

**Table 1:** Soil physicochemical properties for rice cultivation.

	pH	OC (g/kg)	TN (g/kg)	TP (g/kg)	TK (g/kg)	HN (mg/kg)	AP (mg/kg)	AK (mg/kg)	NH_4_^+^–N (mg/kg)	NO_3_^−^–N (mg/kg)	NO_2_^−^–N (mg/kg)
P104	5.90 ± 0.02 c	29.63 ± 0.66 a	2.86 ± 0.02 a	0.74 ± 0.02 b	13.53 ± 0.28 b	266.93 ± 3.93 a	1.21 ± 0.04 c	98.67 ± 1.51 a	4.73 ± 0.30 a	1.32 ± 0.06 a	0.08 ± 0.01 a
P206	5.97 ± 0.02 b	29.22 ± 0.80 a	2.78 ± 0.03 bc	0.72 ± 0.02 b	13.53 ± 0.15 b	254.03 ± 1.68 b	1.34 ± 0.04 b	93.02 ± 1.54 b	3.21 ± 0.17 b	0.82 ± 0.04 c	0.11 ± 0.01 b
P309	6.01 ± 0.03 b	29.03 ± 0.23 a	2.82 ± 0.04 ab	0.79 ± 0.02 a	14.51 ± 0.24 a	263.34 ± 3.46 a	1.17 ± 0.03 c	92.35 ± 1.10 b	4.66 ± 0.27 a	0.56 ± 0.04 d	0.11 ± 0.00 a
P302	6.24 ± 0.03 a	29.34 ± 0.60 a	2.74 ± 0.03 c	0.82 ± 0.02 a	13.70 ± 0.07 b	266.30 ± 5.02 a	1.49 ± 0.04 a	96.58 ± 2.10 a	3.67 ± 0.17 b	1.10 ± 0.07 b	0.11 ± 0.00 a

Data are means ± standard error. Different lowercase letters indicate significant differences (*p* < 0.05) (n = 3).

## Data Availability

The original contributions presented in this study are included in the article and Supplementary Material. Further inquiries can be directed to the corresponding author.

## Supplementary Materials

The following supporting information can be downloaded at: https://www.mdpi.com/article/10.3390/microorganisms13122789/s1, Figure S1: Rarefaction curves for ASVs of bacteria (A), fungi (B), and nitrogen-fixing bacteria (C) in rice rhizosphere soil; Figure S2: Box plot of α-diversity of nitrogen-fixing bacteria; Figure S3: Volcano plot of differential metabolite changes in rice root systems across varieties under positive ion mode; Figure S4: Volcano plot of differential metabolite changes in rice root systems across varieties under negative ion mode; Table S1: The top five metabolites with the most significant differences among various rice varieties.
