# Supplementary material for: Differential Assembly of Rhizosphere Microbiome and Metabolome in Rice with Contrasting Resistance to Blast Disease
Source: Microorganisms. 2025 Dec 8;13(12):2789. doi: 10.3390/microorganisms13122789 (PMC12735870; doi:10.3390/microorganisms13122789)
Supplement: Supplementary file 1 [file microorganisms-13-02789-s001.zip › microorganisms-4003058-supplementary.pdf]

Supplementary Materials

Method

UHPLC-MS/MS analyses were performed using a Vanquish UHPLC system (Thermo Fisher, Germany) coupled with an Orbitrap Q ExactiveTM HF mass spectrometer or Orbitrap Q ExactiveTM HF-X mass spectrometer (Thermo Fisher, Germany) at Novogene Co., Ltd. (Beijing, China). Samples were injected onto a Hypersil Goldcolumn (100×2.1 mm, 1.9μm) using a 12-min linear gradient at a flow rate of 0.2 mL/min. The eluents for the positive and negative polarity modes were eluent A (0.1% FA in water) and eluent B (methanol). The solvent gradient was set as follows: 2% B, 1.5 min; 2-85% B, 3 min; 85-100% B, 10 min; 100-2% B, 10.1 min; 2% B, 12 min. The Q ExactiveTM HF mass spectrometer was operated in positive/negative polarity mode with a spray voltage of 3.5 kV, a capillary temperature of 320°C, a sheath gas flow rate of 35 psi, and an auxiliary gas flow rate of 10 L/min. The S-lens RF level was set to 60, and the auxiliary gas heater temperature was set to 350°C.

Table

Table S1 The top five metabolites with the most significant differences among various rice varieties.

Group	Metabolite Name	
	Positive	Negative
P104 vs P206	Benzyl ferulate	Avermitilol
	S-(p-Azidophenacyl)glutathione	1H,1'H-2,2'-Disulfanediyl-bis-imidazole
	Comazaphilone F	6-deoxy-6-sulfo-D-gluconic acid
	Dodecylamine	N6-Carboxymethyllysine
	Septenine	N-(E-4-coumaroyl)-aspartate
P104 vs P302	L-Asparagine	(-)-Hydroxycitric acid
	N-(3-Indolylacetyl)-L-Alanine	inosine
	3-hydroxy-6H,7H,8H,9H,10H-cyclohexa[c]chromen-6-one	2-O-Feruloyltartronic acid
	L-Glutamine	6-Amino-4-(4-phenoxyphenylethylamino) quinazoline
	Gingerenone A	Buturon
P104 vs P309	H-Pro-Val-OH	6-deoxy-6-sulfo-D-gluconic acid
	O-Succinyl-L-homoserine	Avermitilol
	lavendustin c	3,4-Dihydroxymandelic acid
	Trideca-5,7,9-trienoylcarnitine	N-(E-4-coumaroyl)-aspartate
	Pubeside B	Bungeiside D
P206 vs P302	L-Glutamine	(-)-Hydroxycitric acid
	L-Asparagine	inosine
	3,3'-Diindolylmethane	2-O-Feruloyltartronic acid
	H-TRP-ASP-OH	6-Amino-4-(4-phenoxyphenylethylamino) quinazoline
	N-(3-Indolylacetyl)-L-Alanine	Buturon
P206 vs P309	Decursinol	celebixanthone
	(-)-Solenopsin A	Bungeiside D
	N,N-Dimethylsphingosine	Tiaprofenic acid
	N,N-Dimethyltetradecylamine-N-oxide	5,6-Dimethoxy-3-(4'-methoxyphenylmethyl)phthalide
	R-Palmitoyl-(2-methyl) Ethanolamide	Naringenin chalcone
P302 vs P309	lavendustin c	Genkwanin
	Kirenol	2-Hydroxyacetophenone sulfate
	L-2-amino-8-hydroxyoctanoate	Benzyl nicotinate
	Canthaxanthin	10-Acetyl-3,7-dihydroxyphenoxazine
	N-Acetylmuramate	Naringenin chalcone

Figures

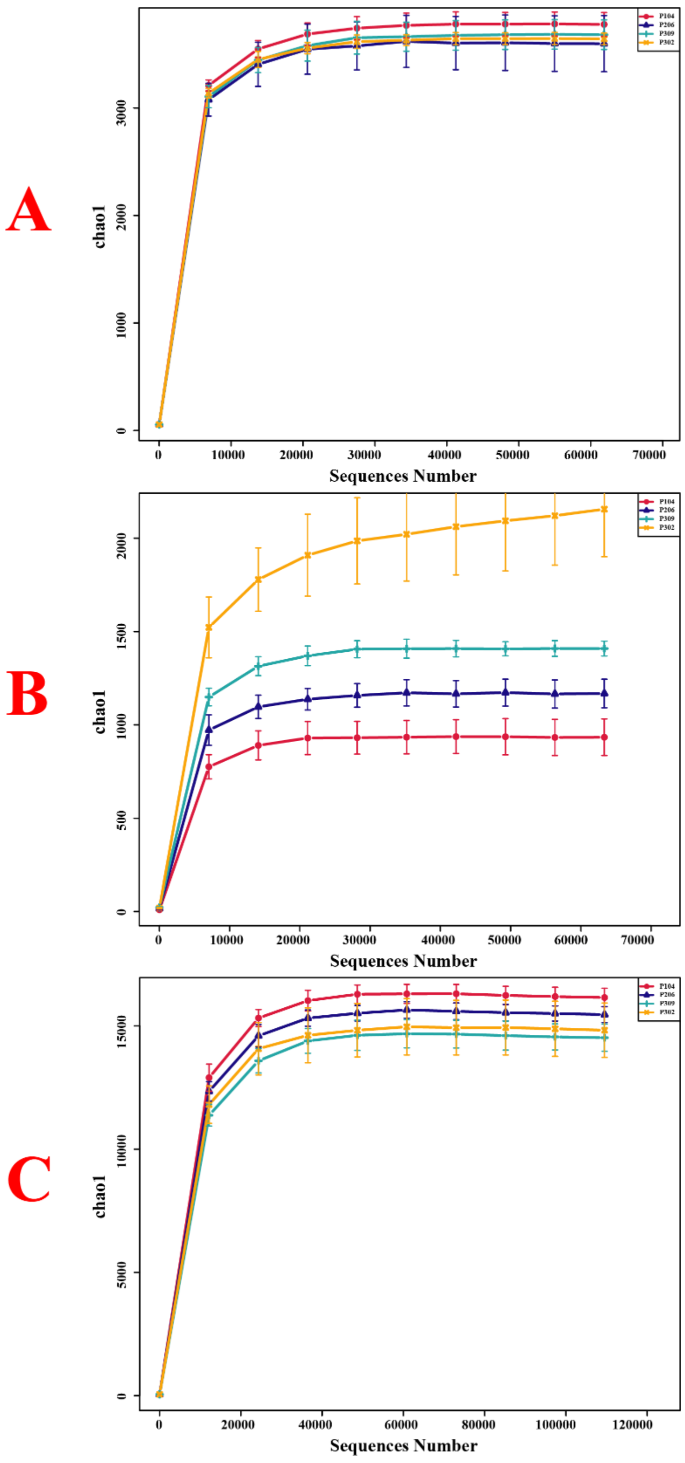

Figure S1 Rarefaction curves for ASVs of bacteria (A), fungi (B), and nitrogen-fixing bacteria (C) in rice rhizosphere soil.

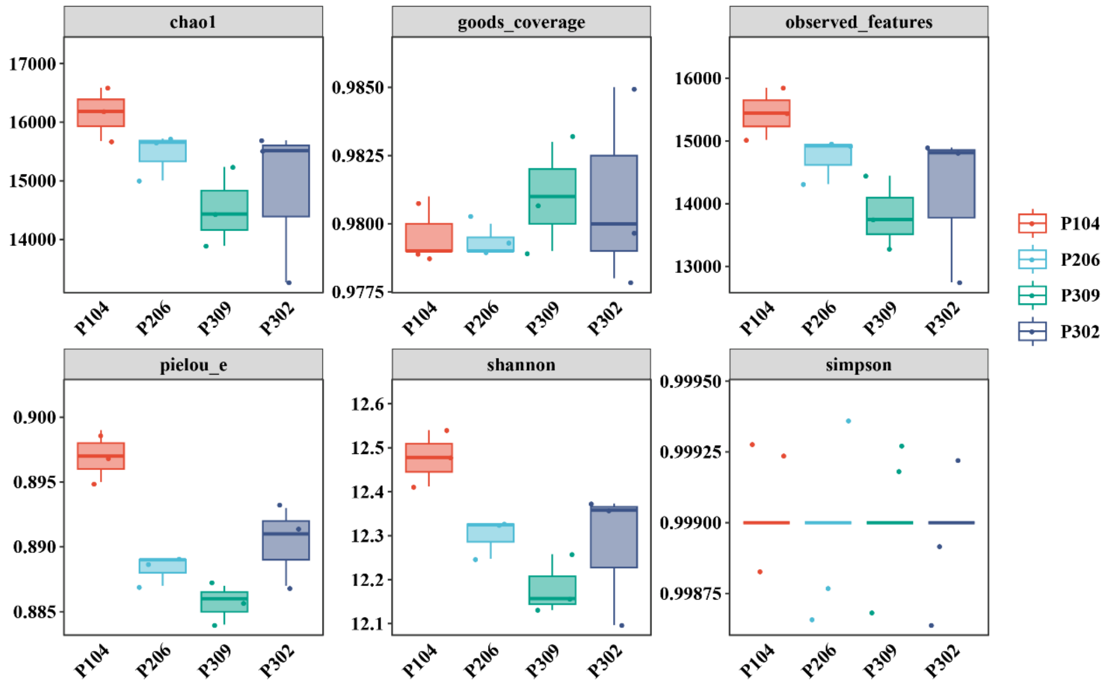

Figure S2 Box plot of α-diversity of nitrogen-fixing bacteria.

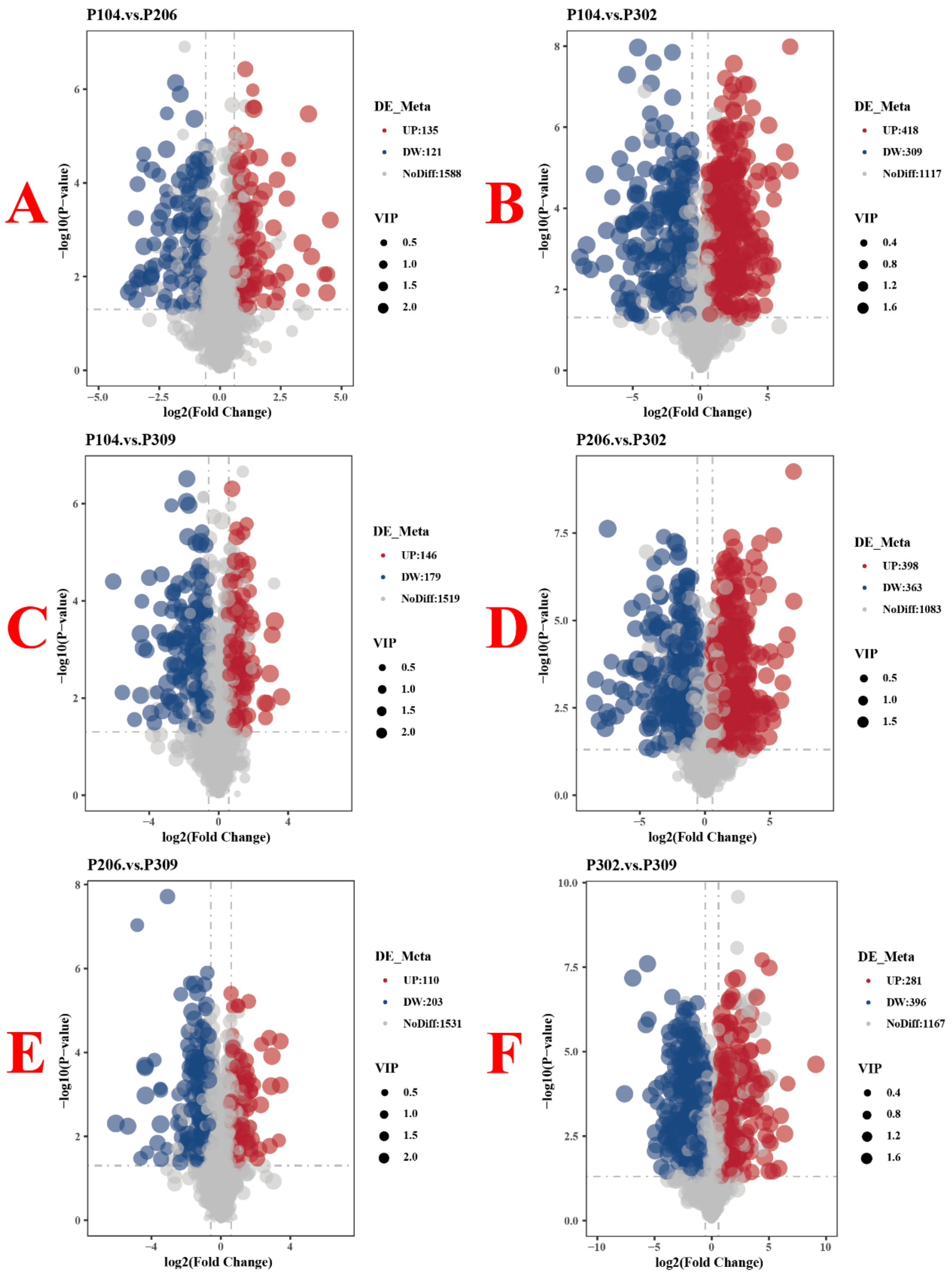

Figure S3 Volcano plot of differential metabolite changes in rice root systems across varieties under positive ion mode.

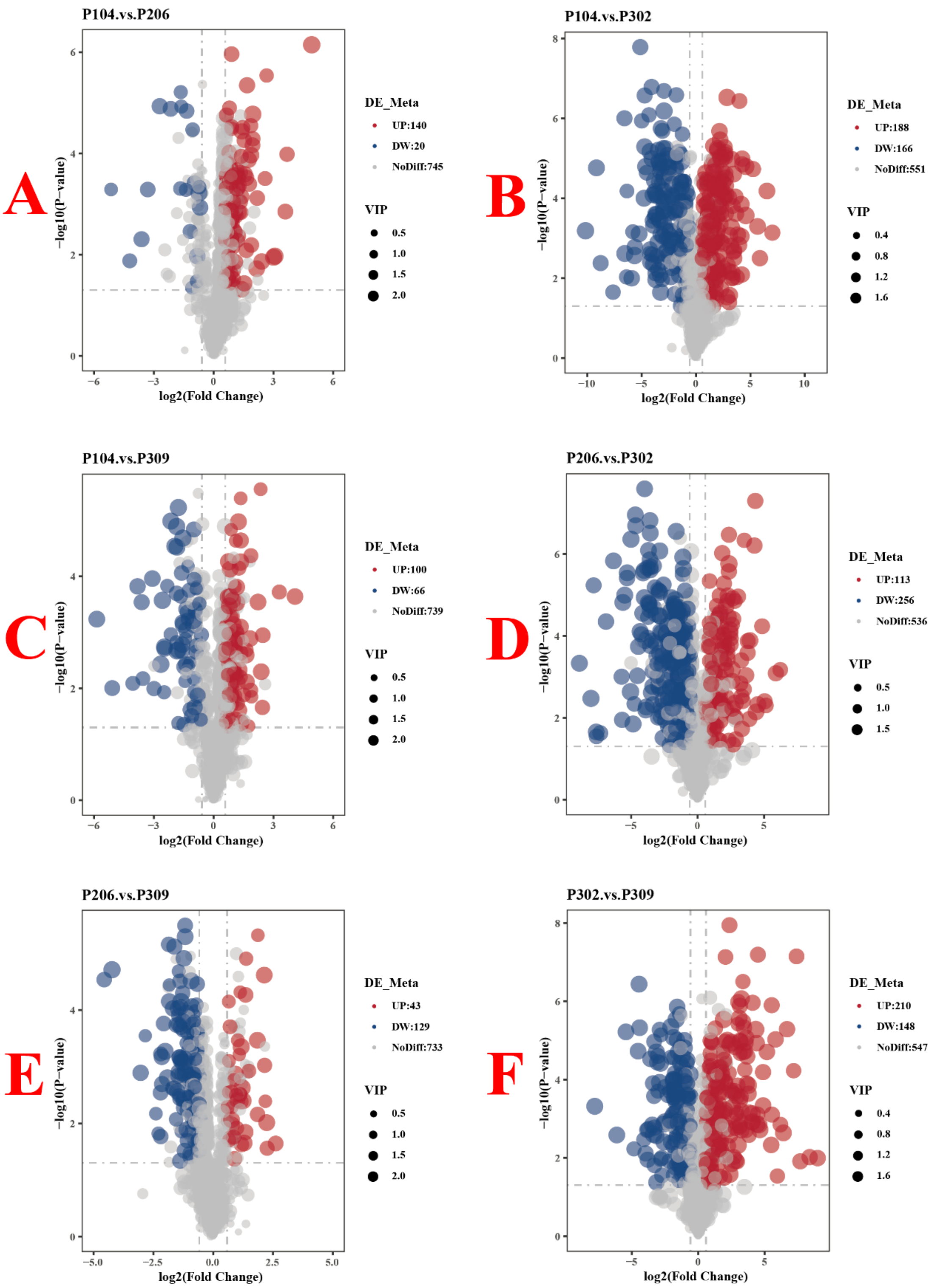

Figure S4 Volcano plot of differential metabolite changes in rice root systems across varieties under negative ion mode.
